# Tuberculous meningitis presenting with unusual clinical features in Nigerians: Two case reports

**DOI:** 10.1186/1757-1626-1-180

**Published:** 2008-09-24

**Authors:** Morenikeji A Komolafe, Taofiki A Sunmonu, Olufunmi A Esan

**Affiliations:** 1 Neurology Unit, Department of Medicine, Obafemi Awolowo University Teaching Hospitals Complex, Ile-Ife, Osun State, Nigeria

## Abstract

**Background:**

Tuberculous meningitis is common in developing countries and accounts for about 7.8% to 14% of all cases of tuberculosis in Nigeria.

**Case presentation:**

Case 1 was a 17-year-old woman who presented with a 3-week history of weakness of the right upper and lower limbs, a 6-hour history of inability to speak and irrational behaviour. She had no remarkable past medical history. Physical examination revealed pyrexia (temperature of 38.2°C) and altered level of consciousness (Glasgow coma score = 7/15). The signs of meningeal irritation were present and she had anisocoria and right spastic hemiparesis. Other aspects of physical examination were normal. Laboratory investigations showed an elevated erythrocyte sedimentation rate, normal cerebrospinal fluid protein and reduced glucose. The brain computed tomography scan showed features in keeping with obstructive hydrocephalus and she was immediately commenced on antituberculous drugs, intravenous steroids and mannitol. She made a remarkable clinical recovery and was discharged home 6 weeks after admission. Case 2 was a 40-year-old man who presented with a 6-week history of headache and fever and a 2-week history of alteration in level of consciousness. There was no history of neck pain and/or stiffness, nausea or vomiting. He had no other remarkable past medical history. He had been placed on various intravenous antibiotics in private hospitals before presentation, with no clinical improvement. Physical examination showed a young man in a coma (Glasgow coma score = 4/15) and febrile (temperature of 38.5°C) with signs of meningeal irritation. The brain stem reflexes were impaired and he had spastic quadriparesis. Further physical examination was essentially normal. The cerebrospinal fluid analysis showed features in keeping with meningeal inflammation and he had a raised erythrocyte sedimentation rate. The brain computed tomography scan showed features in keeping with obstructive hydrocephalus. He was placed on antituberculous drugs and intravenous steroids but despite this his clinical condition deteriorated and he died on the sixth day after admission.

**Conclusion:**

Late presentation of tuberculous meningitis is not rare in Nigerians and we report two cases of tuberculous meningitis that presented late to our health care facility. This report is intended to make clinicians aware of the unusual clinical presentations of tuberculous meningitis.

## Background

It is estimated that over one-third of the world's population has been infected with *Mycobacterium tuberculosis*, and that over 70,000 individuals have tuberculous meningitis (TBM) [1]. In many areas of Africa and Asia, the annual incidence of tuberculosis (TB) infection for all ages is approximately 2% and about 15% to 20% of these cases occur in children. TBM complicates about 1 in 300 of every untreated cases of TB globally, and in Nigeria it accounts for about 7.8% to 14% of all cases of TB [2,3]. Nigerian patients may present late to the hospital and may show atypical clinical and laboratory features [4] which may be misdiagnosed, potentially leading to a high rate of morbidity and mortality in these patients. In this article we report two patients with TBM who presented late to our hospital after receiving treatments from various health facilities with no clinical improvement. This report is intended to increase clinician awareness of late and atypical presentation of TBM.

## Case presentation

### Case 1

A 17-year-old Nigerian woman of Yoruba ethnic origin had been managed for non-specific abdominal pain in the previous 18 months but later defaulted from clinical follow-up. She presented to our unit with a 3-week history of weakness of right upper and lower limbs and a 6-hour history of inability to talk and irrational behaviour. There was associated anorexia, vomiting, fever, neck stiffness and photophobia. She had no seizures or blurring of vision. There was no history suggestive of respiratory, cardiac or urinary abnormalities. There was no personal or family history of hypertension, diabetes mellitus or sickle cell disease. There was no history of blood transfusion, she was not sexually active and her gynaecological history was normal.

On admission, she was pyrexic (temperature of 38.2°C) and a neurological examination revealed an unconscious patient with a Glasgow coma score (GCS) of 7/15 (E_2_V_2_M_3_). She had neck stiffness, and Brudinzki's and Kernig's signs were present. The pupils were bilaterally dilated (6 mm) and were unresponsive to light. There was no papilloedema but she had right facioparesis (upper motor neurone type) and right spastic hemiparesis. Apart from tachycardia (pulse rate of 152 beats per minute), other aspects of physical examination were essentially normal.

Initial investigations showed an erythrocyte sedimentation rate (ESR) of 112 mm/hour (Westergren method). Cerebrospinal fluid (CSF) examination showed normal CSF protein (40 mg/dl) and reduced CSF glucose (1.8 mml/l). The random blood glucose (RBG) was normal (5.2 mml/l) and the CSF to blood glucose ratio was reduced (< 0.5). The CSF microscopy, culture and sensitivity were negative and no acid-fast bacilli were isolated from the CSF. The retroviral screening was negative and chest X-ray showed right middle and lower zone consolidation. A brain computed tomography (CT) scan showed moderate dilatation of all ventricles with associated visualizations of the temporal horns of the left lateral ventricle in keeping with early obstructive hydrocephalus. There was also meningeal enhancement with contrast injection.

The patient was started on antituberculous drugs, that is, rifampicin, isoniazid, ethambutol pyrazinamide and intravenous steroids. Mannitol was also given as adjuvant therapy to relieve the raised intracranial pressure. By the fourth week of admission the patient had made excellent clinical response to antituberculous drugs; her GCS had improved to 15/15 and she had regained full muscle power globally. She was placed on pilocarpine and instructed by the ophthalmologists to wear dark spectacles because of her dilated pupils.

### Case 2

A 40-year-old Nigerian man of Yoruba ethnic origin presented with 6-week history of throbbing and generalized headache and high-grade continuous fever. Two weeks before presentation he developed an altered level of consciousness which was initially confusion and irrational behaviour. He lapsed into unconsciousness a week before presentation. He had lost a considerable amount of weight in the 2 weeks before presentation; however, there was no history of cough, chest pain or night sweating. He had no renal, cardiac or gastro-intestinal symptoms. There was no personal or family history of hypertension, diabetes mellitus or sickle cell disease. He had no history of risk factors for retroviral illness. He had been treated in various private hospitals with no clinical improvement. On admission the patient was chronically ill-looking, cachectic, pale and febrile (temperature of 38.5°C). The neurological examination revealed an unconscious young man with GCS of 4/15 (E_1_V_2_M_1_) with neck stiffness but absent Kernig's and Brudzinski's signs.

There was anisocoria with a left pupil diameter of 4 mm and a right pupil diameter of 2 mm, and both were unreactive to light. The brain stem reflexes were present but motor examination showed global loss of muscle bulk with spastic quadriparesis (power grade 2 globally). Chest examination showed tachypnoea (respiratory rate of 22 cycles per minute)) and widespread transmitted sounds in the chest. Other aspects of physical examination were normal.

**Figure 1:**
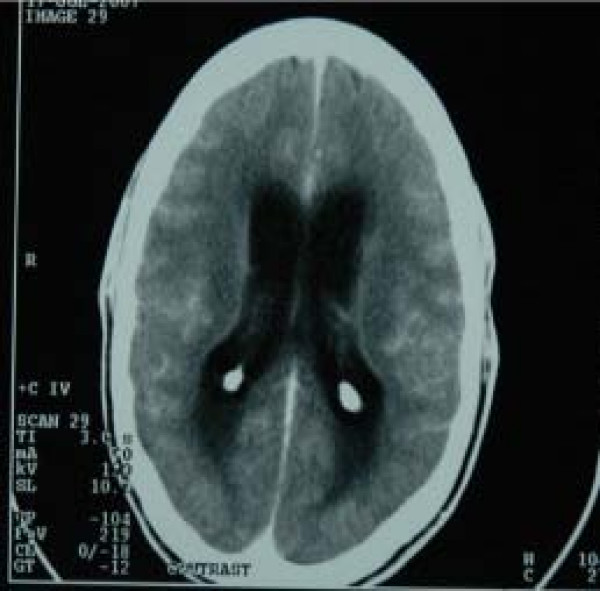
Brain computed tomography scan of case 2 showing dilated ventricles and calcification of choroids plexus.

Initial investigations showed an ESR of 100 mm/hour (Westergren method). The CSF analysis showed CSF protein of 87 mg/dl and CSF glucose of 70 mg/dl. RBG was 106 mg/dl and the CSF to RBG ratio was greater than 0.5. CSF showed numerous red blood cells per high-power field and less than five white blood cells (WBCs) per high-power field. The serum electrolyte, urea and creatinine showed mild azotaemia with urea of 12.1 mmol/l and creatinine of 127 μmol/l while other electrolyte parameters were within normal ranges. Retroviral screening, performed on two occasions, was negative. The haemogram revealed a packed cell volume of 39% and WBC count was 11,000/mm^3 ^with neutrophilia. A brain CT scan showed features in keeping with obstructive hydrocephalus. There was enhancement around the periventricular regions with sulcal effacement (Figure 1).

**Figure 2:**
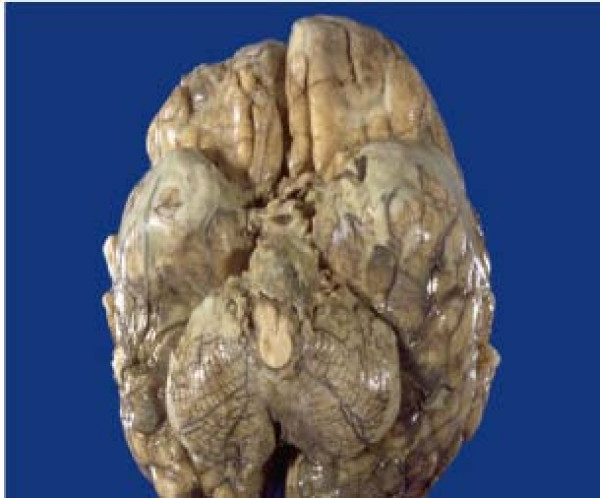
**The gross pathological findings of the brain of case 2 at autopsy showing gelatinous exudates at the base of the brain**. There is also associated brain oedema and congestion.

The patient underwent surgery (ventriculostomy) to relieve the hydrocephalus on the second day of admission and about 200 to 250 ml of CSF was drained over the following 2 days, but his clinical condition deteriorated and he died on the sixth day after admission in the intensive care unit of the hospital. The autopsy study that was carried out showed gelatinous exudates at the base of the brain with severe oedema (Figure 2). The histology of the smear of the exudates showed granulomatous inflammation with Langerhans cells.

## Discussion

TBM begins as a primary infection of the lungs (through inhalation) or rarely following the ingestion of infected milk [5]. The bacteria may spread over several weeks to infect the regional lymph nodes from where bacteraemia may occur, and by haematogenous spread they invade the meninges or the brain parenchyma forming the Rich focus where they remain dormant for many years. With aging or the development of immunosuppression the Rich focus may be re-activated in the central nervous system causing meningitis, meningoencephalitis or cerebral tuberculoma, depending on the initial site of the Rich focus in the brain.

In TBM there is formation of gelatinous exudates which settle at the base of the brain causing central nervous pathologies via cerebral vasculitis (causing infarction or haemorrhage), blockage of CSF pathways (hydrocephalus) and entrapment of cranial nerves. The early symptoms of TBM are usually constitutional, for example fever (low or high grade), malaise, headache, anorexia and recent weight loss. Later the patient may present with alteration in the level of consciousness (confusion to frank coma). The patient may also present with hemiparesis, aphasia, multiple cranial nerve palsies, visual loss, seizures (focal or generalized) or with choreiform limb movement disorders. Usually the late symptoms develop with the onset of complications. The history of onset of illness is usually greater than 3 weeks and in a study among Nigerian patients, the mean duration of illness before presentation was 3.7 weeks. Other unusual presentations such as generalized myoclonus, rigidity, hiccoughs, retention of urine, gait ataxia and hearing impairment were documented in some patients [4].

Investigations that may help in the diagnosis of TBM include full blood count and ESR, which may typically show leukopenia and/or normal WBC count, but leukocytosis and neutrophilia have been reported in some patients with TBM. There may be anaemia with elevated ESR (as in all of the cases reported). Electrolyte urea and creatinine may show hyponatraemia due to development of the syndrome of inappropriate antidiuretic hormone secretion (SIADH) in about 45% of cases. In about 10% of patients the urinalysis may show WBCs without significant bacteriuria, that is, sterile pyuria [6]. CSF was clear in 64%, turbid in 30% and xanthochromic in 8% while spontaneous clotting occurred in 4% of patients in a study in Nigeria [4]. CSF cell count may range from 30 to 1000 WBC/mm^3^, usually a mixture of lymphocytes and neutrophils, and predominantly polymorphonuclear pleocytosis occurs in 15% of patients. CSF glucose is usually lower than normal in 85% of cases while CSF protein may be elevated, ranging from 60 mg/dl to 1 g/dl (see [7]). A Nigerian study documented normal protein in about 6% of patients, and acid-fast bacilli may be isolated from the CSF in 5% to 30% of patients [4].

The tuberculin skin test may be negative in some patients with co-morbid immunosuppressive illness, but immunocompetent patients usually produce positive reactions to the tuberculin test. Electroencephalographic studies often show diffuse slowing, but in cases of cerebral infarction there may be focal slowing. Neuroimaging analysis using brain CT scan with contrast, or magnetic resonance imaging with gadolinium, is generally abnormal showing meningeal enhancement consistent with meningeal inflammation. Areas of infarction and haemorrhage may also be seen in cases of TBM, while patients with late complications may show hydrocephalus (as in both of the cases reported here). Neuroimaging may also show the presence of intracerebral tuberculoma. Meningeal calcification may be obvious on skull X-ray while chest X-ray may show pneumonic process, adenopathy, fibronodular changes, cavitations and pleural effusion if there is associated tuberculous involvement of the lungs. Brain biopsy may be performed at surgery in selected patients and specimens subjected to histopathological studies, Ziehl Nielson stain, fungal studies and culture. The patient should also be screened for immunodeficiency states such as retroviral illness, diabetes mellitus and so on. The unusual clinical and laboratory features such as the presence of high-grade pyrexia (in both of our cases), the presence of normal CSF protein (case 1), normal CSF glucose (case 2) and presence of normal CSF microscopy culture and sensitivity (in both of our cases) may lead to misdiagnosis or late diagnosis of TBM with the attendant high rate of morbidity and mortality.

Multiple drug treatment is required in the management of TBM and the drugs should adequately cross the blood-CSF barrier to achieve a therapeutic concentration in the CSF. The first-line drugs are isoniazid, rifampicin, pyrazinamide, streptomycin and ethambutol while second line anti-TB drugs that could be used include ofloxacin, ciprofloxacin, capreomycin, kanamycin, cycloserine, amikacin, clofazimine and rifabutin. The treatment duration is for 9 to 12 months but this could be extended to 18 to 24 months if there is poor treatment response. If multidrug resistance occurs, second-line drugs should be used. In an Egyptian study, drug resistance in CSF *M. tuberculosis *was low [8]; resistance to isoniazid was 10%, ethambutol was 7%, and rifampicin was 3%; no CSF isolate was multidrug resistant in that study. The incidence of CSF multidrug resistance is higher in children, young adults, patients in developing countries and patients infected with HIV.

The adjunctive therapies include intravenous steroids, for example dexamethasone and oral steroids (prednisolone), usually given in the first 1 to 2 months of therapy to relieve the features of raised intracranial pressure while patients with hydrocephalus will benefit from surgical intervention (ventriculoperitoneal or ventriculo-atrial shunt procedure).

About 50% of TBM patients die and 15% may survive with permanent neurological deficit, while 35% may achieve a full clinical recovery or have minimal sequelae [9]. The clinical stage at the time of presentation is the single most important predictor of clinical outcome, and two recent studies have shown that age, stage of disease, presence of cranial nerve deficit, presence of SIADH, abnormality of electroencephalography, abnormality of motor-evoked potentials and low GCS were associated with poor clinical outcome [10].

## Conclusion

Late presentation of TBM is not rare in our environment. The presence of fever, headaches, recurrent seizures lasting more than 2 to 3 weeks, poor response to conventional antibiotics and the presence of focal neurological signs such as hemiplegia, aphasia and multiple cranial nerve palsies should raise suspicions of TBM in a patient who has the symptoms and signs of meningeal irritation. Also, the CSF findings may be atypical, and the presence of obstructive hydrocephalus should further raise the suspicion of the attending physician for TBM.

## Abbreviations

CSF: cerebrospinal fluid; CT: computed tomography; ESR: erythrocyte sedimentation rate; GCS: Glasgow coma score; RBG: random blood glucose; SIADH: syndrome of inappropriate antidiuretic hormone; TB: tuberculosis; TBM: tuberculous meningitis; WBC: white blood cell.

## Competing interests

The authors declare that they have no competing interests.

## Authors' contributions

MAK conducted the literature review and carried out the review of the patients' medical records. TAS participated in preparation of the manuscript and review of the patients' medical records. OAE participated in the review of patients' medical records. All authors read and approved the final manuscript.

## Consent

Written informed consent was obtained from the patient or their next of kin for publication of these case reports and any accompanying images. A copy of the written consent is available for review by the Editor-in-Chief of the journal.
